# Optimisation of X-Rays Imaging Techniques for the Assessment of Joint Space

**DOI:** 10.5334/jbsr.1447

**Published:** 2018-02-07

**Authors:** Paolo Simoni

**Affiliations:** 1 “Reine Fabiola” Children’s University Hospital, 15, Avenue Jean Joseph Crocq, 1020, Brussels, BE

**Keywords:** X-rays, Multidetector Computed Tomography/standards, Radiation protection, Arthritis, Rheumatoid/diagnostic imaging, Magnetic resonance imaging, Patellofemoral joints, Cartilage, Bone, Humans, Cadavers, Adults

## Introduction

X-ray (XR) imaging techniques, including plain radiography and computed tomography arthrography (CTA) are widely used in clinical settings to assess the joint space and the mineral components of near the joint space, namely the subchondral bone plate and the subchondral bone in both degenerative and inflammatory joint diseases [1,2].

The wide availability, low cost, limited acquisition time, paucity of contraindication and direct visualization of the mineral components of the bone-cartilage unit make XR a reliable tool in musculoskeletal imaging, despite the well-established superiority of magnetic resonance imaging (MRI) for the evaluation of the whole joint and the adjacent tissues in osteoarthritis (OA) and in inflammatory joint disease [2,3]. While MRI is often used as a second line imaging technique or in research, XR techniques are widely used in the routine clinical setting [4,5,6,7,8].

Using MRI, a better spatial resolution is obtained by increasing the acquisition time. Likewise, XR techniques require a higher dose of exposure to obtain a higher level of spatial resolution. On conventional XR, multiple views of the joint are required to better analyse the different sectors of the joint space [9]. Conventional tomography is no longer used because the high radiation burden, especially after the advent of computed tomography [10]. Tomographic views with a lower radiation dose can now be obtained if the XR table is equipped with a digital technique called tomosynthesis [11]. The radiation burden is specifically relevant when imaging joints near organs with high radiation sensitivity such as gonads, thyroid and breast [12,13,14]. Despite the advances in computed tomography and the wide use of CTA in clinical practice, there is no systematic *ex vivo* and *in vitro* study in literature to optimize the dose for joints near radiation-sensitive organs. Using XR imaging techniques to assess the joint space allows an optimization of the dose without loss of information [13,14].

On XR, the current strategy to reduce the radiation dose is to use a single high standardized and reproducible view (e.g. the Lyon schuss view for the internal femoro-tibial joint) in order to measure a single linear estimate (i.e. the minimum joint space width (mJSW)). This estimate has been proven to correlate with the degree of degeneration of the whole joint [15].

Such a measurement has been only validated for the Hip and knee joints [15,16]. As mentioned above, a possibility for a more detailed study of the joint space by thin slices is now possible using tomosynthesis [11]. The dose delivered by tomosynthesis to obtain 1 mm thick slices is comparable to that of standard XR [17].

## General Aim of the Investigation

Three articles [18,19,20] were designed to optimize these three main techniques: XR, tomosynthesis and CTA, in different clinical settings to assess the joint space changes in both degenerative and inflammatory disease.

## X-rays

**Figure 1:**
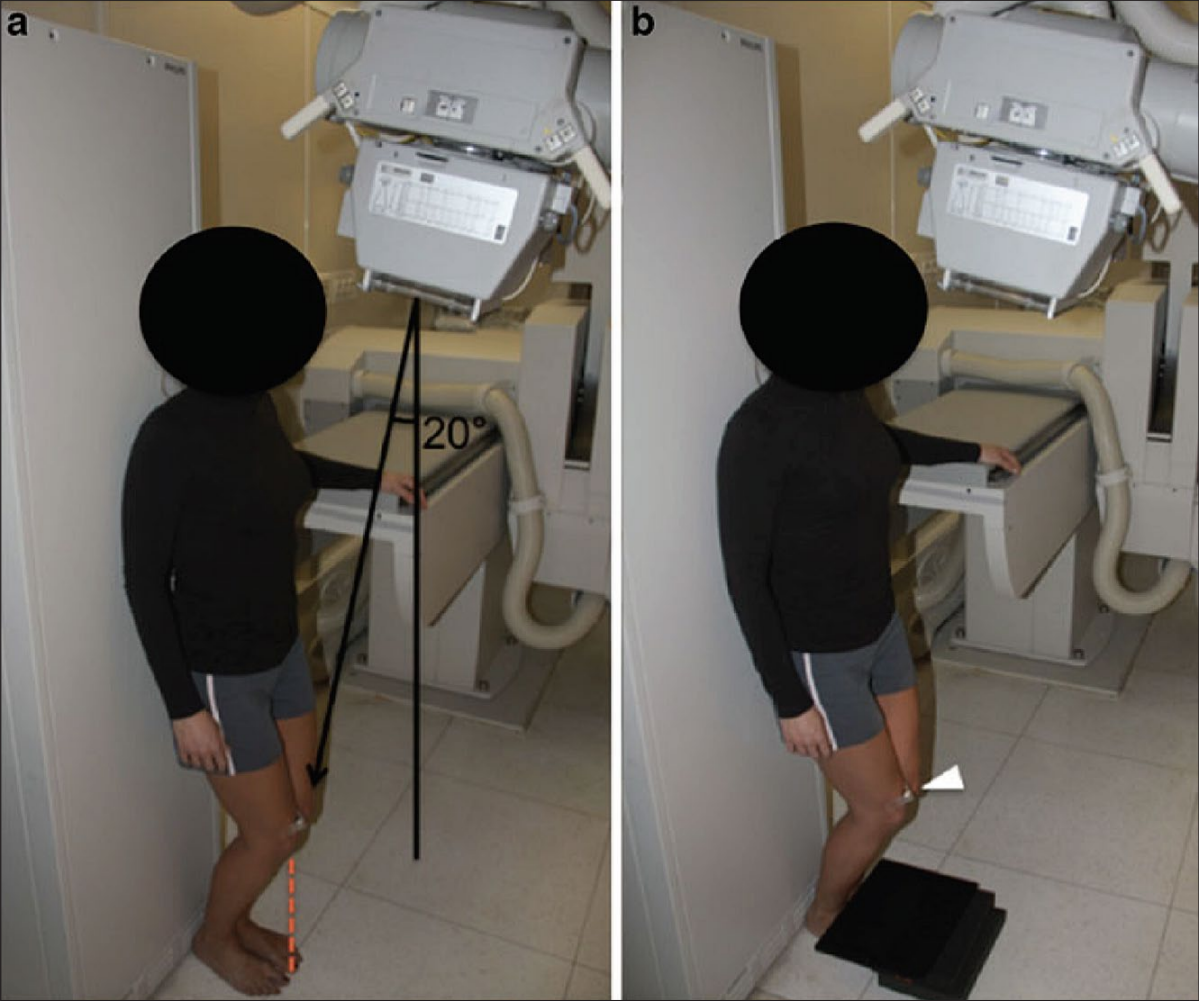
Standing *skyline* radiograph. **(a)** The subject stands against a flat surface with the back, hip, and heel in a coplanar position. The anterior aspect of the patella is beneath the tip of the corresponding big toe (dashed red line). The X-ray beam is angled 20° toward the patient and centred on a point about 2 cm posterior to the anterior aspect of the patella. **(b)** The cassette is positioned parallel to the floor on a piece of foam rubber, which adapts itself to the underlying feet. A 25-mm iron ball (white arrowhead) was positioned at the anterior aspect of the right knee in order to correct for magnification [18].

**Figure 2:**
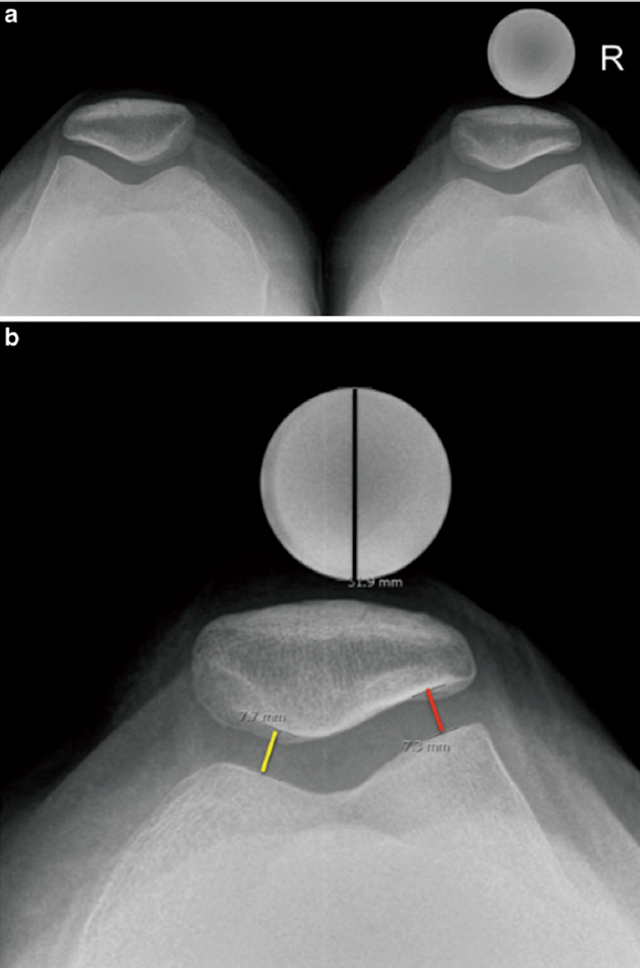
**(a)** Standing *skyline* view of the knees. The 25 mm iron ball, used to correct for magnification, is positioned at the anterior aspect of the centre of the patella. **(b)** Measurements of the medial patellofemoral joint (yellow segment), lateral patellofemoral joint (red segment), and iron ball (black segment) [18].

The aim of this article was to validate and to optimize a new radiographic *skyline* view of the patello-femoral joint. We proposed a variant of a weight-bearing *skyline* view of the femoro-tibial joint (Figure 1). This technique allows performing a standard XR of both patello-femoral joint at a flexion of about 40°. This position increases the pressure on the internal part of the patello-femoral joint, positioning the centre of the patella just in front of the middle third of the patella [21] (Figure 2). This view may reveal a patello-femoral joint space narrowing of the patello-femoral joint, frequently located in this region [22].

**Figure 3:**
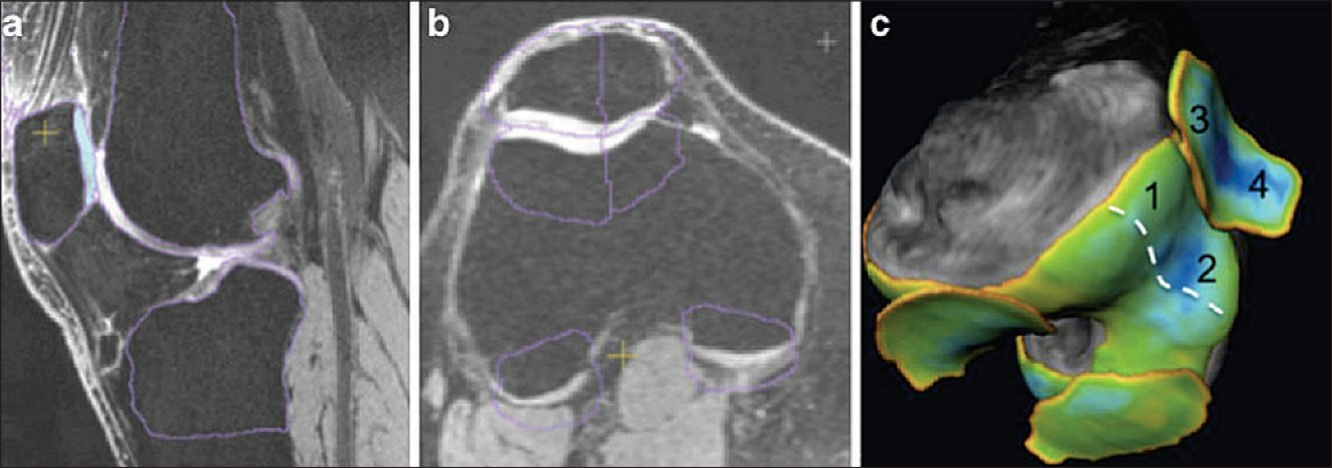
qMRI. **(a)** Sagittal view showing segmented bones and cartilage of patella, tibia and femur. **(b)** Axial image illustrating automated placement of subregional boundaries delimiting left and right facets of the patella and lateral andmedial trochlea. **(c)** 3D image with the mapping of the mean cartilage thickness; the mean thickness of four different subregions was used for the study: medial trochlea (1), lateral trochlea (2), medial patellar facet (3), and lateral patellar facet (4). The mean cartilage thickness of the medial patellofemoral joint was considered as the sum of 1 + 3; similarly, the mean cartilage thickness of the lateral patellofemoral joint was considered as the sum of 2 + 4 [18].

The technique described in the article [18] showed a very high inter-observer agreement (interclass correlation, ICC) as high as 0.9, maximal coefficient of variation of 8.25% at the test retest-analysis, and a statistically significant correlation compared to the estimates of the quantitative MRI (q-MRI). In addition, the measurement of the m-JSW of the patella-femoral joint is significantly correlated to the mean cartilage thickness at qMRI (r = 0.81, p < 0.0001 for the medial patello-femoral compartment and r = 0.71, p < 0.0001 fort the lateral patello-femoral compartment) (Figure 3).

## Tomosynthesis

**Figure 4:**
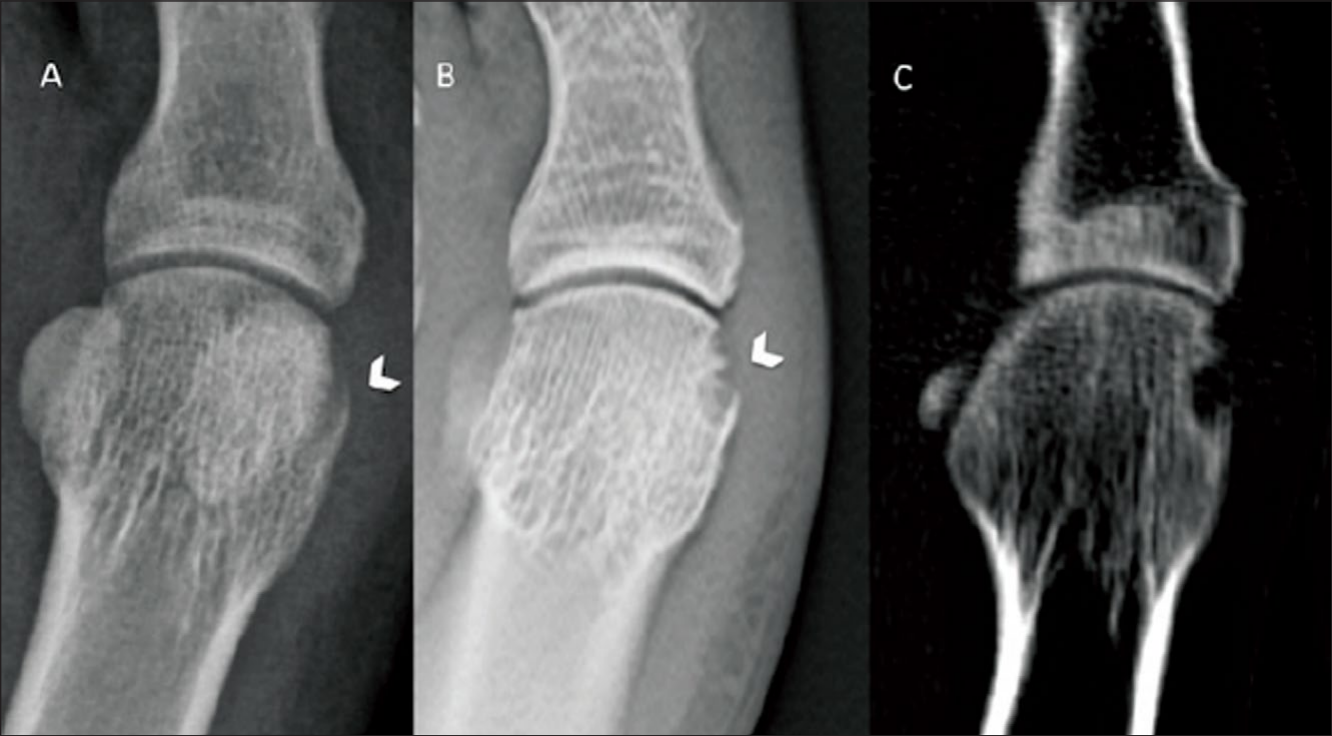
**(a)** A large marginal bone erosion of the medial aspect of the head of the of the first metatarsal bone (white arrowhead) is not visible on standard radiographs but **(b)** It is well visible on tomosynthesis (white arrowhead) and **(c)** It is confirmed by computed tomography (white arrowhead).

In this second article [19], we evaluated the diagnostic performance of tomosynthesis for the detection of joint erosions of the forefoot in patients suffering from clinically proven Rheumatoid Arthritis (RA). Joint-bone erosions are key features in the early diagnosis and follow-up of Rheumatoid Arthritis in clinical settings and in research [23]. Unfortunately, standard X-rays, which are still recommended as a baseline and for RA follow-up, have a very low sensitivity for detecting bone erosions (24% in the Døhn study [24]). MRI has a lower sensitivity to detect bone erosions in patients with RA compared to CT (64–90%) [24,25]. Regarding ultrasound, a large meta-analysis demonstrated that it is equivalent to MRI for the detection of bone erosions in patients with inaugural and established RA [24]. Tomosynthesis is a promising imaging modality for the detection of bone erosions in RA, which can be performed alternatively or in parallel to the standard radiography without a significant increase in the dose and cost of the exam [7,11,17]. A previous study by Canella et al. [26] demonstrated that tomosynthesis detected significantly more erosions in the hands and wrists than radiography in patients with RA (sensitivity increase up to 20%). More recently, Aoki et al. [27] showed that tomosynthesis is superior to radiography and almost comparable to MRI for the detection of bone erosions in the hand and wrist in patients with RA. Our study is the first to compare tomosynthesis to conventional radiography for the detection of bone erosions in the forefoot in patients with established RA (Figure 4). The study of the forefoot is of prime importance in assessing the severity of RA; it makes it possible to complete the radiographic score for the bone erosions of Sharp-van der Heijde [28]. In addition, a recent study has shown that bone erosions are correlated more than joint space narrowing to the degree of functional impotence of the joint in patients with RA [29].

This study [19] enabled some interesting observations. First, tomosynthesis detected a similar number of erosions and resulted in a Sharp-van der Heijde score comparable to those of the CT. On the other hand, the number of erosions and the Sharp-van der Heijde score were statistically higher on CT and X-ray tomosynthesis. Although the Sharp-van der Heijde score has been validated only for radiographies [30], our results suggest that tomosynthesis may provide an assessment of bone erosion that is similar to that of the CT for tomosynthesis. On tomosynthesis, as for CT and X-rays, the number of erosions is strongly correlated with the Sharp-van der Heijde score. This observation may suggest that the size and type of bone erosions seen are the same in all three techniques. Third, tomosynthesis has sensitivity 14% higher than radiographs to show the bone erosions of the forefoot; it can therefore be preferred to conventional radiography for the follow-up of RA. Our results are consistent with the observations of Canella et al. [26], who reported a higher sensitivity of tomosynthesis of 23.7% compared to radiography (77.6% versus 53.9%). Nevertheless, in our study we observed specificity significantly lower than that reported by Canella [26] for X-ray compared to tomosynthesis (81% and 75% respectively in our study and 92% and 89.9% respectively in the Canella study [26].

This difference can have several explanations. On the one hand, our observations showed a higher sensitivity of the radiography compared to the study of Canella [26], which could explain a decrease in the specificity of the radiography. On the other hand, our study differs from the study of Canella [26] with respect to the anatomical site, the mean duration of the disease and the mean age of the patients. Third, radiation doses of conventional radiography and tomosynthesis in the study of the appendicular skeleton are negligible compared with natural radiation (2–3 mSv) [26]. The dose received at the patient’s skin for a tomosynthesis study is similar to that of two conventional radiographs using dorso-plantar and oblique views (0.42 mGy for radiography and 0.56 mGy for tomosynthesis).

## CTA

CTA is still widely used to evaluate the joint hip pathology and cartilage integrity as an alternative to arthrography by magnetic resonance [31,32,33,34,35]. In hip CTA, gonad irradiation makes the problem of radiation burden critical, especially in young patients [14].

**Figure 5:**
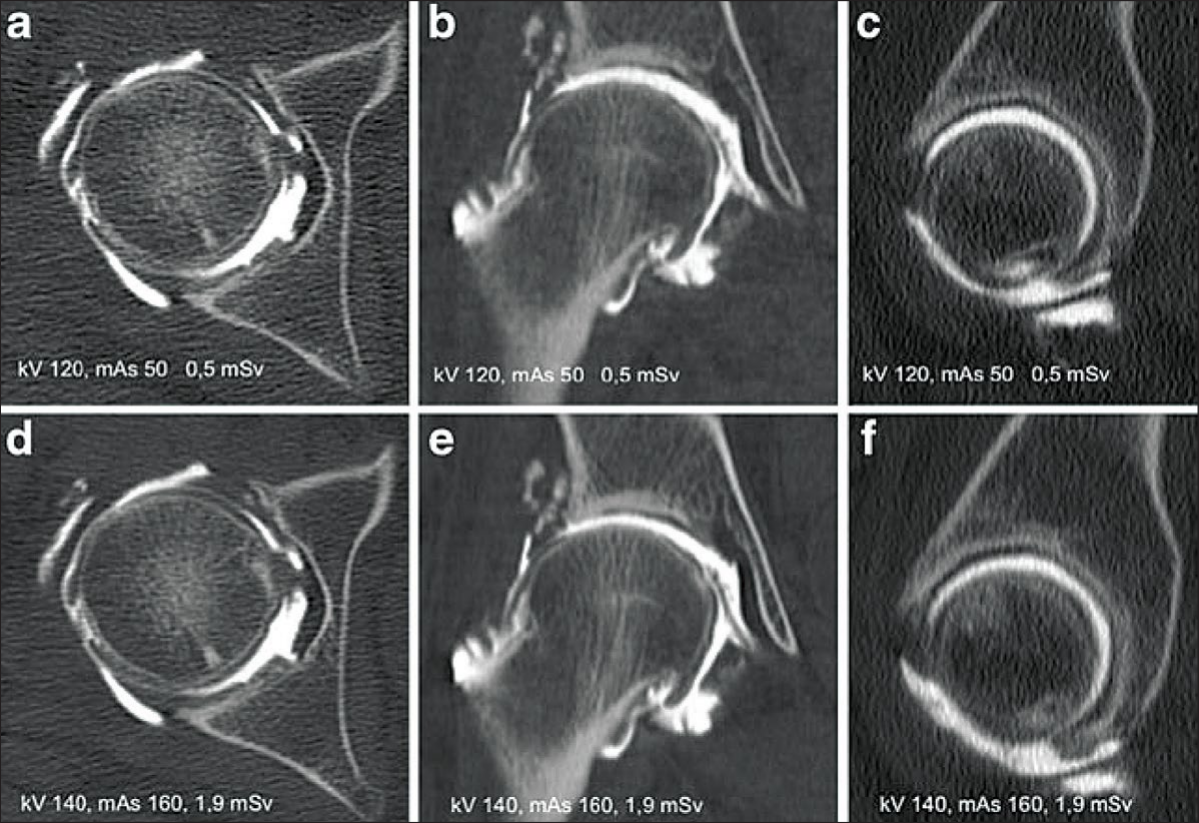
CTA of the hip joint on a patient with a body mass index of 28 kg/m^2^ and acquisition using 120 kVp and 50 mAs. **(a)** Axial, **(b)** Coronal, **(c)** Sagittal multiplanar reconstruction (MPR) and axial **(d)** and Coronal **(e)** and Sagittal **(f)** Reformats obtained with the routine dose of 140 kVp and 160 mAs. Note that even if the noise is higher in the images obtained with the lowest dose (a, b, c), the depiction of the contrast-cartilage and cartilage-subchondral bone plate interfaces is optimal compared with the high-dose standard protocol (d, e, f). Cartilage defect in the upper pole of the femoral head, visible in the coronal reformatted images (b, e), is well depicted by the low-dose protocol (b) without substantial difference from the high-dose protocol (e).

The introduction of the multi-detector CT induced an increase in the radiation load, lessened by different dose modulation techniques [36]. After the introduction of multidetector CT scanners, the literature offers no attempt to optimize the acquisition parameters in the new generation of scanners [14]. Alternatively, iterative reconstructive techniques with *filtered back projection* provide diagnostic images with diminished doses but have not been applied to the CTA in systematic studies [13,37]. Recently, Subhas et al. [38] attempted to optimize the CTA as a variant of the kV and the concentration of intra-articular contrast agent but without reducing the total irradiation dose. Our first study was the first to optimize the value of kV and mAs in the arthritis of the hip for the visualization of the cartilage and the subchondral bone plate. Our study yielded a number of interesting results. First, in vitro and in cadaver, the increase in kV decreased the attenuation value of intra-articular contrast agent, cartilage, and subchondral bone plate. The variation in tissue density with kV is complex. For example, a material with a low atomic number (Z) may be less important than water when the photons are of low energy. Under these conditions, the photoelectric effect predominates. At higher energies, the same tissues are more attenuating than water, because the Compton effect predominates. For materials such as iodine (Z = 53), when the energy of the photons increases, the attenuation decreases because the X-ray interactions with these materials are dominated by the photoelectric effect (inversely proportional to the cube of photon energy) [36,37,39]. Moreover, for iodized contrast, the attenuation is maximal at energy values close to 80 kV due to the energy interaction with the K orbitals of iodine [36,37]. After injection of contrast agent, the cartilage is impregnated with contrast and its change in attenuation can be explained by the presence of iodine [38]. Second, in the phantom and cadaver, the variation of tube current did not show a statistically significant variation in cartilage density (p = 0.06) or subchondral bone (p = 0, 61). The measured attenuation is generally not affected by tube current, but is strictly dependent on kV [39]. Third, on phantom and cadavers, there was a strong influence of kV on the Contrast-to-Noise Ratio (CNR) contrast-cartilage interfaces (p < 0.0001) and the subchondral-cartilage interface (p < 0.0001). The variations in tube current had less effect on the CNR of the contrast-cartilage interface (p = 0.024) and on the subchondral cartilage bone interface (p = 0.011). Indeed, the reduction of the kV leads to a reduction of the CNR. For example, a voltage reduction of 120 to 80 kV reduces the delivered dose by a factor of 2.2 but also increases the noise by a factor of 2 [39]. Similarly, the reduction in tube current causes an increase in the noise of the image (the value of the noise being the promotional inverse to the square root (mA/s)). Fourth, the minimum CNR to obtain a contrast-cartilage interface was at least 4.41; while the minimum CNR for a cartilage-subchondral bone plate interface was 0.4. This shows that the contrast-cartilage interface requires more contrast than the cartilage-subchondral interface to be diagnostic quality. Fifth, the 120 kV/50 mAs protocol was selected from all theoretical combinations based on the lowest dose of radiation (0.5 mSv effective dose). These results are consistent with the literature recommendations for kV. In fact, a 120 kV/50 mAs protocol is sufficient and it can be easily reproduced on all CT devices (Figure 5).

## Conclusion

Despite the growing interest of MRI as a reference imaging method for studying joint space in both osteoarthritis and chronic inflammatory diseases, XR imaging techniques are still widely used in clinical practice because of their low costs and large availability. They allow a direct visualization of subchondral bone plate and of the subchondral bone, components of the bone cartilage unit, which cannot be assessed by MRI. These XR imaging techniques need to be optimized. In our work, we first propose a new technique for the acquisition of the patello-femoral minimal internal and external patello-femoral joint space. This value is strongly correlated with the measurement of the mean thickness of the patello-femoral cartilage calculated on quantitative MRI. We then explored the use of tomosynthesis (digital tomography technique that can be performed on some standard X-ray tables) for the detection of marginal forefoot erosions in patients with rheumatoid arthritis. Finally, we optimized the in vitro and ex vivo a CTA protocol for the hip in order to reduce by 75% the radiation dose delivered to the patient, without compromise in the visualization of the cartilage and the subchondral bone plate.
